# Impact of numeracy on understanding of prostate cancer risk reduction in PSA screening

**DOI:** 10.1371/journal.pone.0190357

**Published:** 2017-12-28

**Authors:** Kevin Koo, Charles D. Brackett, Ellen H. Eisenberg, Kelly A. Kieffer, Elias S. Hyams

**Affiliations:** 1 Section of Urology, Department of Surgery, Dartmouth-Hitchcock Medical Center, Lebanon, New Hampshire, United States of America; 2 Section of General Internal Medicine, Department of Medicine, Dartmouth-Hitchcock Medical Center, Lebanon, New Hampshire, United States of America; University of Zurich, SWITZERLAND

## Abstract

Prostate-specific antigen (PSA) screening for prostate cancer in men of average risk remains controversial. Patients’ ability to incorporate risk reduction data into their decision-making may depend on their numeracy. We assessed the impact of patients’ numeracy on their understanding of the risk reduction benefits of PSA screening. Men attending a general internal medicine clinic were invited to complete a survey. Four versions of the survey each included a three-item numeracy test and PSA risk reduction data, framed one of four ways: absolute (ARR) versus relative risk reduction (RRR), with or without baseline risk (BR). Respondents were asked to adjust their perceived risk of prostate-cancer mortality using the data presented. Accuracy of risk reduction was evaluated relative to how risk data were framed. Among a total of 200 respondents, a majority incorrectly answered one or more of the numeracy items. Overall accuracy of risk adjustment was only 20%. Accuracy varied with data framing: when presented with RRR, respondents were 13% accurate without BR and 31% accurate with BR; when presented with ARR, they were 0% accurate without BR and 35% accurate with BR. Including BR data significantly improved accuracy for both RRR (P = 0.03) and ARR groups (*P* < 0.01). Accuracy was significantly related to numeracy; numeracy scores of 0, 1, 2, and 3 were associated with accuracy rates of six, five, nine, and 36 percent, respectively (*P* < 0.01). Overall, numeracy was significantly associated with the accuracy of interpreting quantitative benefits of PSA screening. Alternative methods of communicating risk may facilitate shared decision-making in the use of PSA screening for early detection of prostate cancer.

## Introduction

Prostate cancer is the most common non-cutaneous cancer in American men and will account for about 27,000 deaths in 2017, the third leading cause of cancer mortality [1]. The use of prostate-specific antigen (PSA) screening for the early detection of prostate cancer in men of average risk remains controversial. Recognizing that screening entails risks and benefits and is a preference-sensitive decision, the American College of Physicians, American Cancer Society, American Society of Clinical Oncology, and American Urological Association have advocated a shared decision-making model between patients and clinicians to meet patients’ goals of care [2–5].

Patient education materials and in-person counseling about PSA screening often cite results from clinical trials to explain the potential effects of screening on cancer mortality. These statistics may take the form of relative risk reduction, absolute risk reduction, odds ratios, or “number needed to treat” [6, 7]. A requirement of shared decision-making and risk communication is that patients are able to incorporate and apply quantitative information in their decision-making. However, patients’ ability to interpret probability and risk data may depend on their numeracy, or facility with quantitative concepts [6, 8, 9].

Population-based studies of health numeracy demonstrate a persistent discrepancy between patients’ skills and the quantitative demands of health-care decisions, such as weighing treatment options or maintaining adherence to therapy. The most recent National Assessment of Adult Literacy found that over half of U.S adults possess “below basic” or “basic” math skills [10]. Furthermore, education is not necessarily a valid proxy for numeracy, as college-educated adults may still struggle with numerical concepts [11, 12].

To date, few studies have directly examined the role of numeracy in men’s decision-making about screening for prostate cancer. We conducted a cross-sectional survey study to assess the impact of patients’ numeracy on their understanding of risk reduction benefits of PSA screening.

## Materials and methods

### Study population

Consecutive men 40–75 years old presenting to a general internal medicine clinic were invited to complete a survey at the time of registration. The inclusion age range was selected to represent a population of men who would be likely to have heard of prostate cancer as a condition and might be considering PSA testing for the purpose of early detection of prostate cancer. Men who had been presented with the survey at a prior visit during the study period were excluded. To ensure anonymity, no identifying patient information was collected, and completed surveys were returned by respondents in sealed envelopes prior to departing the clinic.

### Survey design

Surveys contained three parts: a three-item numeracy instrument assessing probability concepts [13]; information about risk reduction in PSA screening for prostate cancer, followed by two questions about PSA screening based on respondents’ perceived risk of prostate-cancer mortality; and, finally, questions regarding demographics, socioeconomic status, educational background, and personal history of PSA testing and prostate cancer.

The numeracy instrument assessed respondents’ familiarity with basic probability. The first item asked respondents how many times on average 1000 flips of a fair coin would come up heads (500 times). The second item asked respondents to convert a percentage to a proportion: in a lottery game in which 1% of players win a $10 prize, how many $10 winners would be expected if 1000 players each bought one lottery ticket (10 winners in 1000). The third item posed the reverse task and asked respondents to convert a proportion to a percentage: in a sweepstakes in which the chance of winning a car is one in 1000, find the percentage of sweepstakes tickets that would win a car (0.1%).

**Fig 1:**
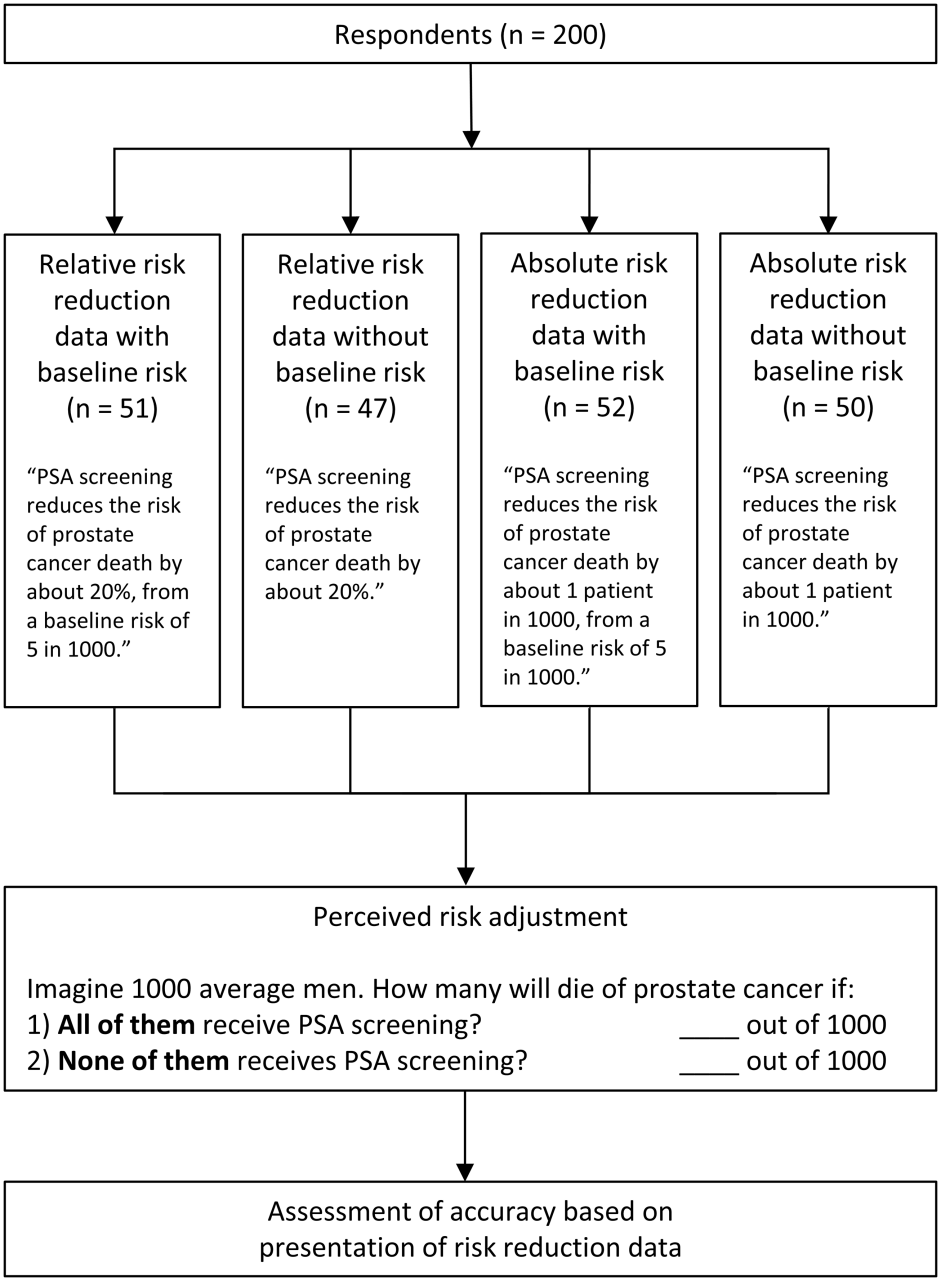
Study design and perceived risk reduction task based on data presentation.

Four versions of the survey were distributed to potential participants in a repeating sequence (A, B, C, D). The survey versions differed only in the way that the risk reduction effect of PSA screening was framed: 20% relative risk reduction from a baseline risk of five in 1000; 20% relative risk reduction without baseline risk; 1 in 1000 absolute risk reduction from a baseline risk of five in 1000; or a 1 in 1000 absolute risk reduction without baseline risk. Based on the data when provided and their own knowledge or preconceived notions, respondents were then asked to calculate the effect of prostate-cancer screening on cancer-related death by estimating how many men among 1000 average men would die of prostate cancer if *all* of them received PSA screening, and how many men among 1000 average men would die of prostate cancer if *none* of them received PSA screening. The relative and absolute risk data were approximated based on results of the European Randomized Study of Screening for Prostate Cancer [14] to simplify the calculation. Fig 1 summarizes the four survey groups and the risk reduction task. S1 File includes the four versions of the survey.

Because there was no existing literature on which to base a power calculation, enrollment of participants continued until a reasonable number was reached to allow for quantitative comparison.

### Analysis of numeracy and accuracy of risk reduction

Respondents’ numeracy was scored as the number of correct answers on the three-item numeracy instrument (range 0–3).

Accuracy of risk reduction was calculated by comparing respondents’ perceived risk of prostate-cancer mortality with PSA screening versus their perceived risk without PSA screening. Because not all surveys contained baseline risk data, accuracy was determined by the change in risk, not the absolute number, such that respondents who over- or underestimated baseline risk could still be considered accurate as long as the risk reduction was applied correctly. Respondents who wrote out calculations without performing them (e.g., “20% of 5” for men with PSA screening) were considered accurate if the calculations were correct.

To differentiate unanswered survey items due to difficulty or skill from refusal to participate in the study, surveys that contained responses to at least 75% of the demographics section (the final section of the survey and the most straightforward to answer) were considered complete and included in the analysis. Unanswered numeracy or risk reduction questions in complete surveys were considered incorrect.

Data were compiled for descriptive analysis. Two-sided comparisons of demographic characteristics and responses to numeracy and accuracy tasks among the four survey groups were performed using the chi-square and Kruskal–Wallis tests and considered significant at *P* < 0.05.

The study was reviewed by the Dartmouth College Committee for the Protection of Human Subjects (#28927) and granted exempt status as minimal risk research.

## Results

Of 336 men who were eligible and invited to participate, 200 returned complete surveys that were included for analysis (60% response rate). Mean age was 60 years (interquartile range 53–68). 91% of respondents had completed at least a high-school education and 53% were employed. About half of the sample reported an annual income of less than $50,000. 51% of respondents had received a PSA test, and 5% reported a personal prostate cancer diagnosis. Respondent characteristics are summarized in Table 1. No significant differences in characteristics were identified among the four survey groups.

**Table 1:** Demographics and clinical history of the study sample.

Characteristics	% (n = 200)
Highest educational attainment	
Did not graduate high school	9.1
Graduated high school	31.7
Graduated college	29.6
Graduated graduate or professional school	29.6
White/Caucasian race	96.5
Employment status	
Employed	52.5
Not employed	6.1
Retired	41.4
Annual income, $1000s	
< $25	20.7
$25–50	23.8
$50–100	28.5
> $100	27.0
Ever received a PSA test	50.5
Ever diagnosed with prostate cancer	5.1

A majority of respondents (56%) incorrectly answered at least one of the three numeracy questions. Of a possible numeracy score of three correct answers, 27% scored 2, 20% scored 1, and 9% answered none of the tasks correctly. The proportion of respondents who correctly answered all three numeracy items did not differ significantly among the four survey groups (range 38–55%).

Study participants found some numeracy tasks more challenging than others. 15% of respondents had difficulty with the basic probability of a coin flip (i.e., 1000 flips of a fair coin would be expected to come up heads about 500 times); incorrect answers ranged from 1 to 950, most commonly 50 or 100. 26% of respondents were unable to calculate 1% of 1000, with 100 being the most common incorrect response. Finally, half of the sample could not convert “1 in 1000” to a percentage; wrong answers were split among 0.001%, 0.01%, and 1%.

**Fig 2:**
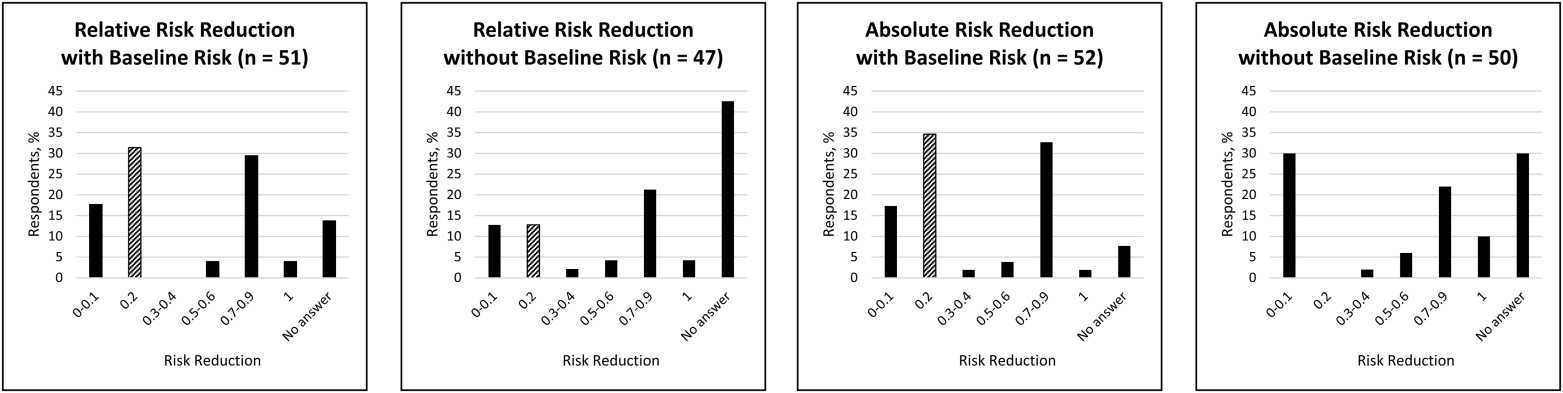
Men’s estimates of the risk reduction benefit of PSA screening. Risk reduction data were presented in four ways: relative risk reduction with and without baseline risk and absolute risk reduction with and without baseline risk. The correct response (0.2) is highlighted with diagonal bars.

Accuracy of perceived risk reduction of prostate cancer mortality was poor at 20% overall. Of the demographic characteristics, only education and income were associated with accuracy. None of the respondents who did not complete high school were accurate, compared with 10% of those who had completed high school, 20% of those who had completed college, and 37% of those who had completed graduate school (*P* = 0.03). By income category, 10% of the respondents who reported an annual income of less than $25,000 were accurate, compared with 26% of those between $25,000 and $50,000, 4% of those between $50,000 and $100,000, and 40% of those greater than $100,000 (*P* < 0.01). Neither PSA testing history nor prostate cancer diagnosis was associated with accuracy. Accuracy was different among the four survey groups based on how the risk reduction data was presented (Fig 2). Respondents who were given relative risk reduction data were 13% accurate when the data was framed without baseline risk and 31% accurate with baseline risk. For absolute risk reduction groups, respondents were 0% accurate when the data were framed without baseline risk and 35% accurate with baseline risk. Including baseline risk data significantly improved accuracy for both the relative risk (*P* = 0.03) and absolute risk groups (*P* < 0.01). In all groups, inaccurate answers to the risk reduction task tended to be numerically greater than the correct response (Fig 2), reflecting respondents’ overestimation of the risk reduction effect, regardless of how the data were presented.

Respondents who were not given baseline risk data were less likely to complete the perceived risk reduction task compared to their counterparts who were provided with these data. This was observed in the “no answer” response for the question about the prostate cancer mortality reduction benefit of PSA screening (S1 Fig) and the question about mortality without screening (S2 Fig). Notably, when not provided with baseline risk, most respondents who did provide an answer tended to dramatically overestimate the mortality rate, with or without screening, by at least an order of magnitude (e.g., greater than 100 deaths per 1000).

Higher numeracy scores were associated with greater accuracy on the risk reduction task across all survey groups. The 36% of respondents who achieved a numeracy score of three correct answers were significantly more likely to perform the task accurately than those with lower numeracy scores (36% with a numeracy score of 3 were accurate compared to 6% with a score of 0, 5% with a score of 1, and 9% with a score of 2) (*P* < 0.01). Compared with a numeracy score of 0, the odds ratio for accuracy for respondents with a score of 3 was 9.5 (*P* = 0.01).

## Discussion

Inadequate health literacy has been linked to poor health outcomes, low-quality health communication, increased and unexpected health-care costs, and the persistence of health disparities among patients with low educational and socioeconomic status [8, 15–19]. As a component of literacy, health numeracy is likely an integral factor in patient education, counseling, and informed decision-making [20]. Low numeracy skills have adverse effects on patients’ grasp of complex quantitative concepts, including probability and risk, and may distort their understanding of risks and benefits of screening and treatment [7, 13, 21].

In this study of men’s ability to adjust perceived risk of prostate-cancer mortality based on PSA risk reduction data, we found that how risk data are presented to patients can have a profound effect on their ability to interpret and apply the data accurately. While relative risk presentations were about as effective as absolute risk presentations with respect to patients’ accuracy of risk adjustment, the inclusion of baseline risk significantly enhanced accuracy. The considerable increases in accuracy in both relative and absolute risk groups suggest that the addition of this baseline risk may help patients better contextualize the quantitative benefits of potential interventions. Of note, a plurality of respondents in the groups not provided baseline risk did not give a valid response to the risk reduction task (“no answer” columns in Fig 2), whereas the proportion of “no answer” was considerably lower in the groups given baseline risk. This may reflect a lower response rate among those respondents with higher numeracy who recognized that baseline risk data was not provided and thus did not attempt the task. Finally, we did not find that absolute risk was superior to relative risk for improving accuracy as prior studies have shown [13, 22, 23]. Absolute risk may nonetheless have advantages over relative risk for communication about screening decisions since relative risk has been suggested to be more likely to motivate initiation of therapy [7]; it was beyond the scope of this study to determine whether there is a similar influence on choosing to undergo screening.

Notably, the risk reduction task was designed such that respondents who applied the risk data appropriately to any baseline prostate cancer mortality rate would be scored as accurate. Thus, the demonstrated effect of framing the data using baseline risk is not likely due only to a more accurate “starting point,” as this should not have affected men’s quantitative capacity to apply the risk reduction data. Instead, the addition of baseline risk improved respondents’ calculation of the change in risk with and without PSA screening—from 0% to 36% in the absolute risk reduction group.

This is important because when men were inaccurate, they tended to overestimate the benefit of screening (Fig 2). For example, in the group that received absolute risk reduction data with baseline risk, nearly as many men believed that PSA screening would reduce prostate cancer deaths by 70–90% as the ones who accurately answered 20%. In contrast to the very small proportion of men who “minimally overestimated” the effect of PSA screening that might be explained by arithmetic errors, the most common answer (27% of respondents) to the risk reduction task “maximally overestimated” the actual benefit of PSA screening. Consistent with prior studies about the public’s enthusiasm for cancer screening in general [24] and overestimation of its benefits [25], these findings suggest that patients may be inherently biased towards the belief that PSA screening is more beneficial than it actually is, and support the need to convey individual risks and accurately define a realistic effect of PSA screening during patient counseling.

The impact of data framing, however, was nonetheless limited relative to respondents’ poor overall accuracy of 20%. The robust relationship between numeracy and accuracy of applying quantitative risk concepts was tempered by the finding that even among the men who attained perfect numeracy scores, about two-thirds of the men were still unable to adjust their perceived risk of cancer mortality appropriately. Respondents with numeracy scores of two or lower fared significantly worse. These results are particularly striking in this highly-educated population in which nearly 60% of respondents had a college degree.

Our findings indicate that even with adequate numeracy, most patients are likely to find concepts of probability and risk challenging. Prior work has suggested that using a denominator of 1000 when presenting risk data, as we did in this study, facilitates greater accuracy [7, 26], but clinical evidence is often presented to patients as percentages or odds ratios between 0 and 1. Risk communication may be complicated by low overall literacy, as poor numeracy has also been associated with low comprehension of simple terminology in prostate disease [27] and unreliable results on the American Urological Association Symptom Score [28]. Taken together, the present findings have implications not only for men deciding whether or not to get a PSA test, but also weighing risk and benefits more broadly, such as the odds of disease-free survival after treatment of localized cancer.

Successful shared decision-making regarding PSA screening, which is recommended by evidence-based guidelines of numerous specialty societies, requires patients and clinicians to consider the best available evidence informed by patients’ goals and preferences [29]. Quantitative data is an inherent part of these discussions, yet complex barriers persist: patients frequently do not have the numeracy skills to absorb and interpret risk statistics [10], and physicians may not, either [30]. Often misconceptions go uncorrected because patients who have difficulty interpreting numerical facts may be embarrassed or ashamed to request clarification [31, 32].

To combat these challenges, several decision aids specific to prostate cancer have been developed to assist in shared decision-making [33, 34], including those that are targeted to high-risk patients, can be used in multispecialty clinics, and assess decisional regret after prostate-cancer treatment [35–37]. Tools that are patient-specific and incorporate validated strategies for visually communicating risk, such as the “incremental risk icon array” in the Prostate Cancer Prevention Trial nomogram [38], may be easier to adopt and more likely to succeed. In the present study, we tested numeracy and accuracy of risk reduction without graphical representations of the risk data, as graphical literacy may be challenging to prostate-cancer patients independent of numeracy [39]. Whether visual methods for risk communication benefit some or all patients with low numeracy in PSA screening discussions should be further explored.

Several limitations should be noted. First, our response rate was 60%, and we cannot account for the characteristics of the patients who declined to participate or specify the reasons they refused. It is also possible that the medical problem for which patients were presenting could have affected the numeracy findings. However, 60% is a reasonable rate of completion for a self-administered survey in a clinic setting, and we did not consider the presenting condition as an eligibility criterion so that the responses would capture a broad, diverse population of male patients. Furthermore, the most likely effect on the findings would be an underestimation of the impact of numeracy assuming those who declined did so due to the challenging quantitative tasks. The similar response rate among the four forms of the survey suggests that study subjects’ decision to participate was not influenced by the version of the survey they received. Although the numeracy instrument was not formally validated, it had the advantages of being a concise, practical assessment, and having precedent in the literature [13]; we elected to include it in the survey in the absence of a more efficient or rigorous option. We also acknowledge that numeracy is connected to overall literacy, and patients who struggle to read would be less able to comprehend the instructions and content of the survey instruments. Our sample also had higher education and income levels—both predictors of higher numeracy—than average U.S. men, likely related to geography of our institution and sampling biases. Still, we found that numeracy was poor overall, underscoring concerns about the computational demands on average men of PSA-screening age. Also, we did not perform a power calculation because of a lack of existing literature on which to base an expectation of statistical difference. While it is thus possible that the differences are due to chance, the results do provide meaningful exploratory insight on the potential interaction between numeracy and decision-making. Finally, while the study population may not reflect the demographic diversity of all U.S. adults, the effect of poor numeracy on patients’ quantitative understanding is likely generalizable.

## Conclusion

Patients’ numeracy was significantly associated with the accuracy of interpreting quantitative benefits of PSA screening. Although accuracy of adjustment in prostate-cancer mortality improved when the presentation of risk reduction data was framed by baseline risk, numeracy as measured by a self-administered survey instrument was poor overall in this population of men. The findings identify a potential problem of low numeracy challenging the shared decision-making process. It is possible that alternative methods of communicating concepts of risk to patients, particularly among those with lower numeracy and literacy, may facilitate shared decision-making in the early detection of prostate cancer.

## Supporting information

S1 File Study survey. Four versions of the survey were distributed to eligible participants in a repeating sequence (A, B, C, D).
(PDF)

S1 Fig Responses by data presentation format to the question, “Imagine 1000 average men. based on the study results and what you know about prostate cancer, how many will die of prostate cancer if all of them receive PSA screening?” The correct answer (4) is highlighted in red.
(TIF)

S2 Fig Responses by data presentation format to the question, “Imagine 1000 average men. based on the study results and what you know about prostate cancer, how many will die of prostate cancer if none of them receives PSA screening?” The correct answer (5) is highlighted in red.
(TIF)
