# Supplementary material for: Impact of numeracy on understanding of prostate cancer risk reduction in PSA screening
Source: PLoS One. 2017 Dec 28;12(12):e0190357. doi: 10.1371/journal.pone.0190357 (PMC5746255; doi:10.1371/journal.pone.0190357)
Supplement: S1 File — Four versions of the survey were distributed to eligible participants in a repeating sequence (A, B, C, D). (PDF) [file pone.0190357.s001.pdf]

Please answer the following questions.
Questions 1–3 are about probabilities, or the chance that something will happen.

Question 1.
Imagine that we flip a normal coin 1000 times. How many times would the coin come up heads in 1000 flips? ____ time(s) out of 1000

Question 2.
In the Big Bucks Lottery, the chance of winning a $10 prize is 1%. How many people would win a $10 prize if 1000 people each buy a single ticket to the Big Bucks Lottery? ____ person(s) out of 1000

Question 3.
In Acme Publishing Sweepstakes, the chance of winning a car is 1 in 1000. What percentage of tickets to Acme Publishing Sweepstakes wins a car? ____ %

Questions 4 and 5 are about a test for prostate cancer.

PSA screening is a way of testing men for the chances of having prostate cancer. Imagine that a hypothetical study showed that on average, PSA screening reduces the risk of prostate cancer death by about 20%, from a baseline risk of 5 in 1000.

Question 4.
Imagine 1000 average men. Based on the study results and what you know about prostate cancer, how many will die of prostate cancer if **all of them** receive PSA screening? ____ out of 1000

Question 5.
Imagine 1000 average men. Based on the study results and what you know about prostate cancer, how many will die of prostate cancer if **none of them** receives PSA screening? ____ out of 1000

Please answer the following questions about yourself.

Age ____ years old

Highest level of education that you completed

- ◯ Did not finish high school
- ◯ Completed high school
- ◯ Completed college
- ◯ Completed graduate school

Race

- ◯ White/Caucasian
- ◯ African-American
- ◯ Hispanic/Latino
- ◯ Asian/Pacific Islander
- ◯ Other

Employment	◯ Employed<br>◯ Unemployed<br>◯ Retired
Yearly income (now or at time of retirement)	◯ Less than $25,000<br>◯ $25,000 to $50,000<br>◯ $50,000 to $100,000<br>◯ More than $100,000
Have you ever had a PSA (prostate-specific antigen) test?	◯ Yes ◯ No ◯ Not sure
Have you ever had prostate cancer?	◯ Yes ◯ No
Do you think every man should have a PSA test?	◯ Yes ◯ No ◯ No opinion

When you have finished, please put this survey in the envelope provided.
Thank you for your participation.

Please answer the following questions.
Questions 1–3 are about probabilities, or the chance that something will happen.

**Question 1.**<br>Imagine that we flip a normal coin 1000 times. How many times would the coin come up heads in 1000 flips?	____ time(s) out of 1000
**Question 2.**<br>In the Big Bucks Lottery, the chance of winning a $10 prize is 1%. How many people would win a $10 prize if 1000 people each buy a single ticket to the Big Bucks Lottery?	____ person(s) out of 1000
**Question 3.**<br>In Acme Publishing Sweepstakes, the chance of winning a car is 1 in 1000. What percentage of tickets to Acme Publishing Sweepstakes wins a car?	____ %

Questions 4 and 5 are about a test for prostate cancer.

PSA screening is a way of testing men for the chances of having prostate cancer. Imagine that a hypothetical study showed that on average, PSA screening reduces the risk of prostate cancer death by about 20%.

**Question 4.**<br>Imagine 1000 average men. Based on the study results and what you know about prostate cancer, how many will die of prostate cancer if **all of them** receive PSA screening?	____ out of 1000
**Question 5.**<br>Imagine 1000 average men. Based on the study results and what you know about prostate cancer, how many will die of prostate cancer if **none of them** receives PSA screening?	____ out of 1000

Please answer the following questions about yourself.

Age	____ years old
Highest level of education that you completed	◯ Did not finish high school<br>◯ Completed high school<br>◯ Completed college<br>◯ Completed graduate school
Race	◯ White/Caucasian<br>◯ African-American<br>◯ Hispanic/Latino<br>◯ Asian/Pacific Islander<br>◯ Other

Employment	◯ Employed<br>◯ Unemployed<br>◯ Retired
Yearly income (now or at time of retirement)	◯ Less than $25,000<br>◯ $25,000 to $50,000<br>◯ $50,000 to $100,000<br>◯ More than $100,000
Have you ever had a PSA (prostate-specific antigen) test?	◯ Yes ◯ No ◯ Not sure
Have you ever had prostate cancer?	◯ Yes ◯ No
Do you think every man should have a PSA test?	◯ Yes ◯ No ◯ No opinion

When you have finished, please put this survey in the envelope provided.
Thank you for your participation.

Please answer the following questions.
Questions 1–3 are about probabilities, or the chance that something will happen.

Question 1.
Imagine that we flip a normal coin 1000 times. How many times would the coin come up heads in 1000 flips? ____ time(s) out of 1000

Question 2.
In the Big Bucks Lottery, the chance of winning a $10 prize is 1%. How many people would win a $10 prize if 1000 people each buy a single ticket to the Big Bucks Lottery? ____ person(s) out of 1000

Question 3.
In Acme Publishing Sweepstakes, the chance of winning a car is 1 in 1000. What percentage of tickets to Acme Publishing Sweepstakes wins a car? ____ %

Questions 4 and 5 are about a test for prostate cancer.

PSA screening is a way of testing men for the chances of having prostate cancer. Imagine that a hypothetical study showed that on average, PSA screening reduces the risk of prostate cancer death by about 1 patient in 1000, from a baseline risk of 5 in 1000.

Question 4.
Imagine 1000 average men. Based on the study results and what you know about prostate cancer, how many will die of prostate cancer if **all of them** receive PSA screening? ____ out of 1000

Question 5.
Imagine 1000 average men. Based on the study results and what you know about prostate cancer, how many will die of prostate cancer if **none of them** receives PSA screening? ____ out of 1000

Please answer the following questions about yourself.

Age ____ years old

Highest level of education that you completed
- ◯ Did not finish high school
- ◯ Completed high school
- ◯ Completed college
- ◯ Completed graduate school

Race
- ◯ White/Caucasian
- ◯ African-American
- ◯ Hispanic/Latino
- ◯ Asian/Pacific Islander
- ◯ Other

Employment	◯ Employed<br>◯ Unemployed<br>◯ Retired
Yearly income (now or at time of retirement)	◯ Less than $25,000<br>◯ $25,000 to $50,000<br>◯ $50,000 to $100,000<br>◯ More than $100,000
Have you ever had a PSA (prostate-specific antigen) test?	◯ Yes ◯ No ◯ Not sure
Have you ever had prostate cancer?	◯ Yes ◯ No
Do you think every man should have a PSA test?	◯ Yes ◯ No ◯ No opinion

When you have finished, please put this survey in the envelope provided.
Thank you for your participation.

Please answer the following questions.
Questions 1–3 are about probabilities, or the chance that something will happen.

Question 1.
Imagine that we flip a normal coin 1000 times. How many times would the coin come up heads in 1000 flips? ____ time(s) out of 1000

Question 2.
In the Big Bucks Lottery, the chance of winning a $10 prize is 1%. How many people would win a $10 prize if 1000 people each buy a single ticket to the Big Bucks Lottery? ____ person(s) out of 1000

Question 3.
In Acme Publishing Sweepstakes, the chance of winning a car is 1 in 1000. What percentage of tickets to Acme Publishing Sweepstakes wins a car? ____ %

Questions 4 and 5 are about a test for prostate cancer.

PSA screening is a way of testing men for the chances of having prostate cancer. Imagine that a hypothetical study showed that on average, PSA screening reduces the risk of prostate cancer death by about 1 patient in 1000.

Question 4.
Imagine 1000 average men. Based on the study results and what you know about prostate cancer, how many will die of prostate cancer if **all of them** receive PSA screening? ____ out of 1000

Question 5.
Imagine 1000 average men. Based on the study results and what you know about prostate cancer, how many will die of prostate cancer if **none of them** receives PSA screening? ____ out of 1000

Please answer the following questions about yourself.

Age ____ years old

Highest level of education that you completed
- ◯ Did not finish high school
- ◯ Completed high school
- ◯ Completed college
- ◯ Completed graduate school

Race
- ◯ White/Caucasian
- ◯ African-American
- ◯ Hispanic/Latino
- ◯ Asian/Pacific Islander
- ◯ Other

Employment	◯ Employed<br>◯ Unemployed<br>◯ Retired
Yearly income (now or at time of retirement)	◯ Less than $25,000<br>◯ $25,000 to $50,000<br>◯ $50,000 to $100,000<br>◯ More than $100,000
Have you ever had a PSA (prostate-specific antigen) test?	◯ Yes ◯ No ◯ Not sure
Have you ever had prostate cancer?	◯ Yes ◯ No
Do you think every man should have a PSA test?	◯ Yes ◯ No ◯ No opinion

When you have finished, please put this survey in the envelope provided.
Thank you for your participation.
