# Supplementary figures and images for: Impact of numeracy on understanding of prostate cancer risk reduction in PSA screening
Source: PLoS One. 2017 Dec 28;12(12):e0190357. doi: 10.1371/journal.pone.0190357 (PMC5746255; doi:10.1371/journal.pone.0190357)

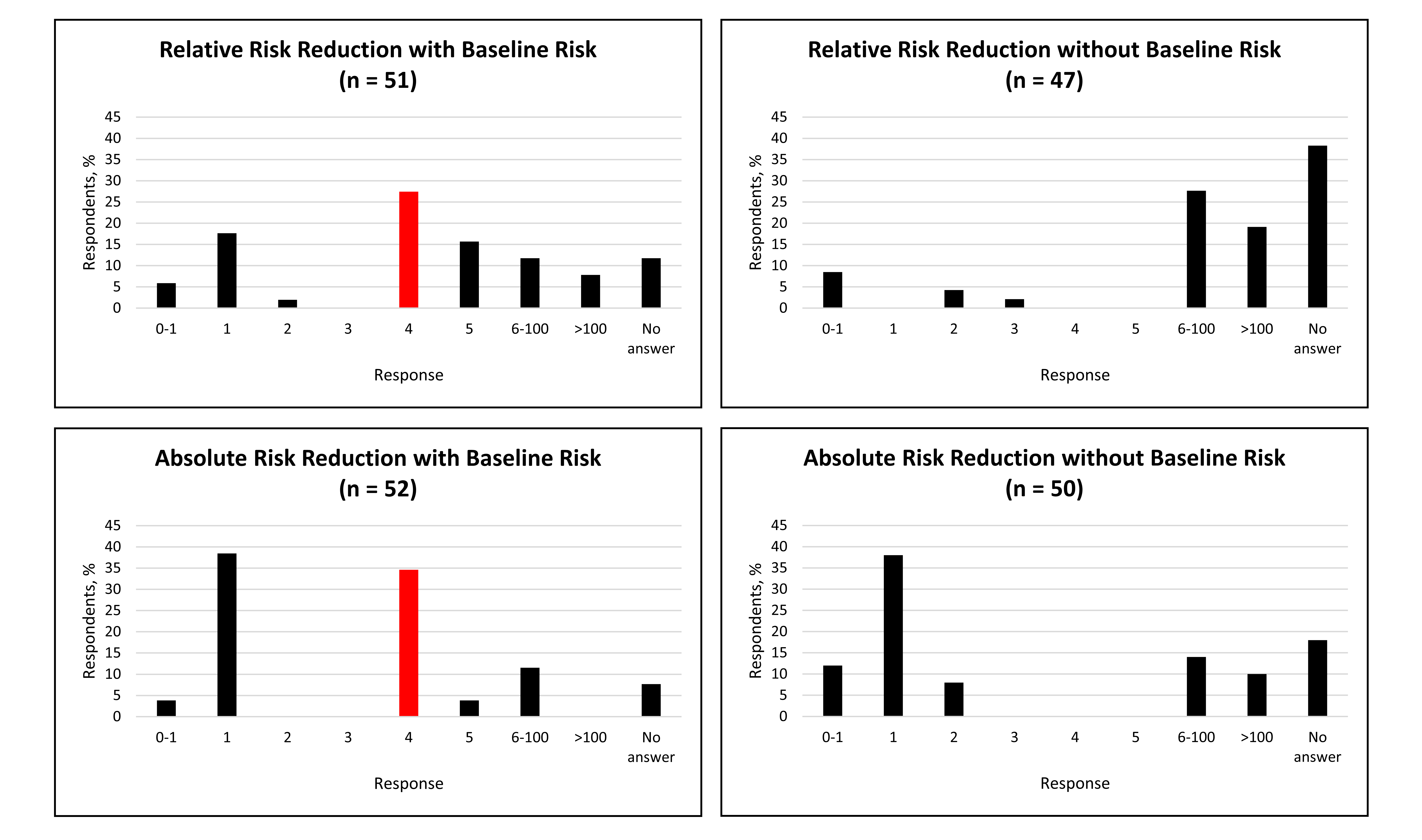

Supplement: S1 Fig — The correct answer (4) is highlighted in red. (TIF) [file pone.0190357.s002.tif]

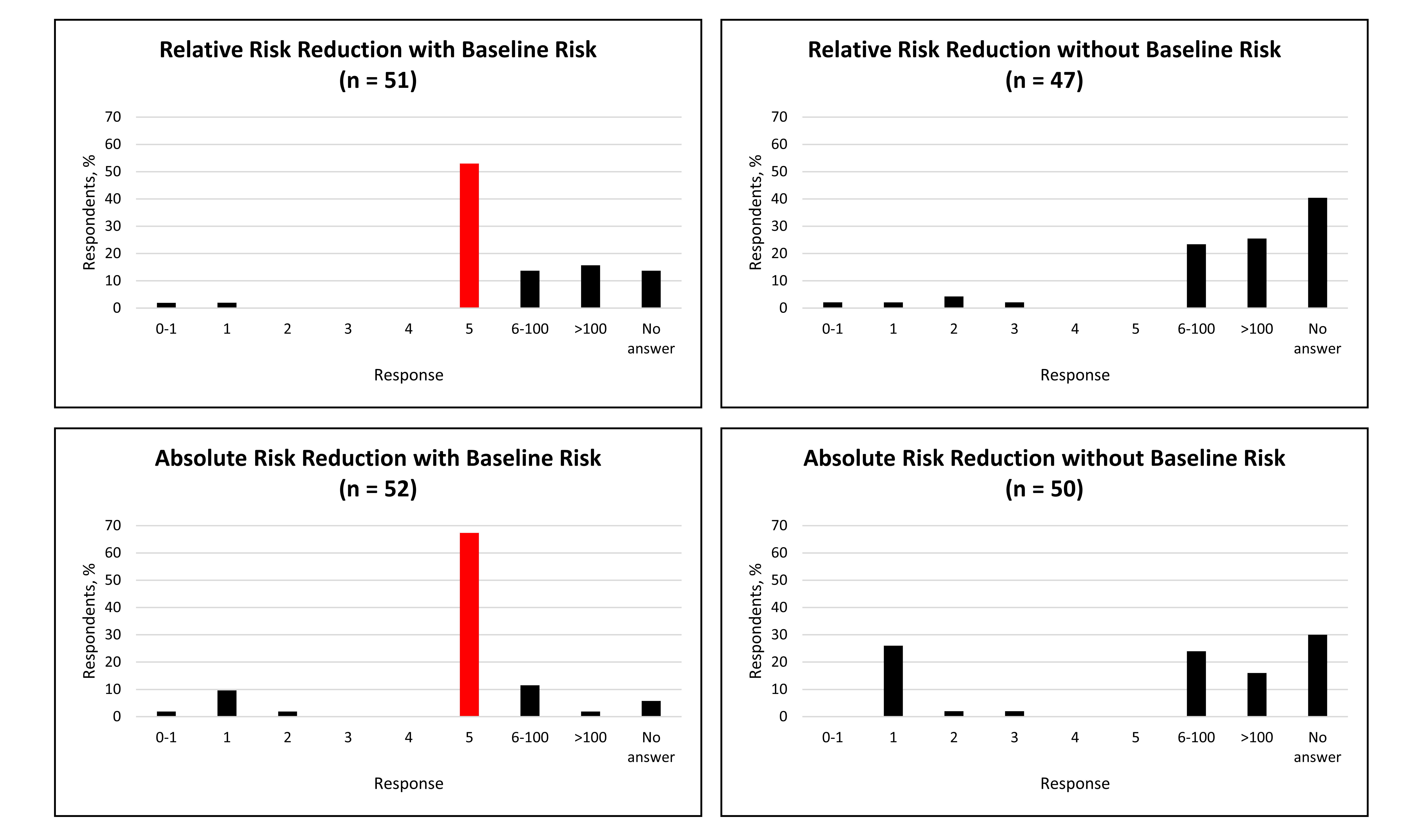

Supplement: S2 Fig — The correct answer (5) is highlighted in red. (TIF) [file pone.0190357.s003.tif]
